# Multi-Dimensional Display of Wang’s Lymph Node Map Using Virtual Bronchoscopic Navigation System

**DOI:** 10.3389/fmolb.2021.679442

**Published:** 2021-06-07

**Authors:** Fen Lan, Yaling Yue, Hong Shen, Hui Shen, Qiyuan Wang, Xiujing Yu, Laijuan Chen, Qin Li, Kopen Wang, Qinghua Liu, Yang Xia

**Affiliations:** ^1^ Key Laboratory of Respiratory Disease of Zhejiang Province, Department of Respiratory and Critical Care Medicine, Second Affiliated Hospital of Zhejiang University School of Medicine, Hangzhou, China; ^2^ Department of Medical Oncology, Handan Central Hospital, Handan, China; ^3^ Department of Respiratory and Critical Care Medicine, Second Affiliated Hospital of Nanjing Medical University, Nanjing, China; ^4^ Department of Respiratory and Critical Care Medicine, Huzhou Central Hospital, Huzhou, China; ^5^ Department of Radiology, Second Affiliated Hospital of Zhejiang University School of Medicine, Hangzhou, China; ^6^ Department of Endoscopic Center, Second Affiliated Hospital of Zhejiang University School of Medicine, Hangzhou, China; ^7^ Division of Pulmonary and Critical Care Medicine, Johns Hopkins University School of Medicine, Baltimore, MD, United States; ^8^ Department of Respiratory and Critical Care Medicine, Shanghai East Hospital, Tongji University, Shanghai, China

**Keywords:** lymph node map, TBNA, mediastinal-hilar lymph node, virtual navigation system, bronchoscopy

## Abstract

**Background:** Transbronchial needle aspiration (TBNA) is a classical technique for diagnosing mediastinal-hilar lymph node enlargement. However, the diagnostic value of conventional TBNA (cTBNA) is limited in small lymph nodes.

**Methods:** Here, we generated an innovative multi-dimensional virtual lymph node map on top of Wang’s lymph node map using a Lungpoint Virtual Bronchoscopic Navigation System.

**Results:** The virtual bronchoscopic navigation (VBN) system was combined with computed tomography (CT) images to generate extrabronchial, endobronchial, sagittal, coronal as well as horizontal views of the 11 intrathoracic lymph node stations and their adjacent tissues and blood vessels. We displayed the specific puncture site of each lymph node station. The 11 stations were divided into four groups: right mediastinal stations, left mediastinal stations, central mediastinal stations and hilar stations.

**Conclusion:** The VBN system provides a precise view of the intrabronchial landmarks, which may increase the diagnostic accuracy of intrathoracic lymph node adenopathy and assist bronchoscopists with practicing TBNA.

## Introduction

Transbronchial needle aspiration (TBNA) is a classical bronchoscopic technique for diagnosing benign and malignant mediastinal-hilar lymph node enlargement. With technological progress, endobronchial ultrasound-guided TBNA (EBUS-TBNA) provides real-time ultrasound imaging, resulting in a higher diagnostic yield than conventional TBNA (cTBNA) and has been widely practiced in clinical settings worldwide (Herth et al., 2004; Bonifazi et al., 2017; Madan et al., 2017). However, even with ultrasound guidance, the diagnostic yield of TBNA varies, largely due to the skill and experience of the operator (Rodriguez de Castro et al., 1997; Hsu et al., 2004). Therefore, whether the operator can accurately locate the target lymph nodes is of great importance, and requires a comprehensive understanding of the anatomy of mediastinal-hilar lymph nodes.

The International Association for the Study of Lung Cancer (IASLC) lymph node map and Wang’s lymph node map are the two most commonly used lymph node maps (Wang, 1994; Rusch et al., 2009). The eighth edition of the IASLC map was introduced in 2009 and most of the lymph node stations are defined according to the positions of blood vessels (El-Sherief et al., 2014). Without ultrasound, it is challenging to identify the blood vessels under bronchoscopy. Wang’s map was proposed in 1994 and the targeted lymph nodes are located by airway landmarks under endoscopic view, which is more practical for practitioners (Wang, 1994).

Electromagnetic navigation bronchoscopy (ENB) was first applied in human subjects in 2006 (Schwarz et al., 2006). Compared with traditional bronchoscopy, ENB improved the diagnostic accuracy for peripheral pulmonary lesions (PPLs) and mediastinal lymph nodes (Gildea et al., 2006). However, the EBN process requires expensive equipment and special training for operators, and therefore it can only be applied in qualified medical centers. On the other hand, virtual bronchoscopy can simulate a three-dimensional view of the bronchi and adjacent structures using. The reported diagnostic yield of virtual bronchoscopic navigation (VBN) system for peripheral pulmonary lesions was 70.9%–76.8% for peripheral pulmonary lesions, that of thin bronchoscopy combined with computed tomography (CT) and VBN was 65.4%–81.6% and that of X-ray fluoroscopy plus VBN was 62.5%–78.7% (Asano et al., 2014). Electromagnetic navigation and virtual navigation could also be used as advanced lymph node localisation systems. Previously, we reported the intrabronchial display of hilar-mediastinal lymph nodes by the SPiN Thoracic Navigation System (Wu et al., 2018). However, only an endobronchial map of mediastinal-hilar lymph nodes was generated, without demonstration of the extrabronchial anatomical position of each lymph node. Here, we further devised a virtual extrabronchial and endobronchial map together with horizontal, coronal and sagittal views on chest CT for each mediastinal and hilar lymph node using the Lungpoint Virtual Bronchoscopic Navigation System, in accordance with the recommended lymph node puncture site in Wang’s map. Therefore, clinicians can gain an overall impression of the three-dimensional (3D) spatial structure of the most frequently punctured lymph nodes, thus improving the accuracy and safety of TBNA.

## Methods

CT images 1 mm thick were acquired and transferred to a workstation where the Lungpoint Virtual Bronchoscopic Navigation System (Broncus Technologies, Inc., Mountain View, CA, United States) automatically generated a 3D model of the airway. We set up 3D markers of the locations of intrathoracic lymph nodes. The extrabronchial anatomical positions of each target lymph node were shown as green spheres. The positions and borders of 11 target lymph nodes were described according to Wang’s lymph node map.

## Results

**FIGURE 1:**
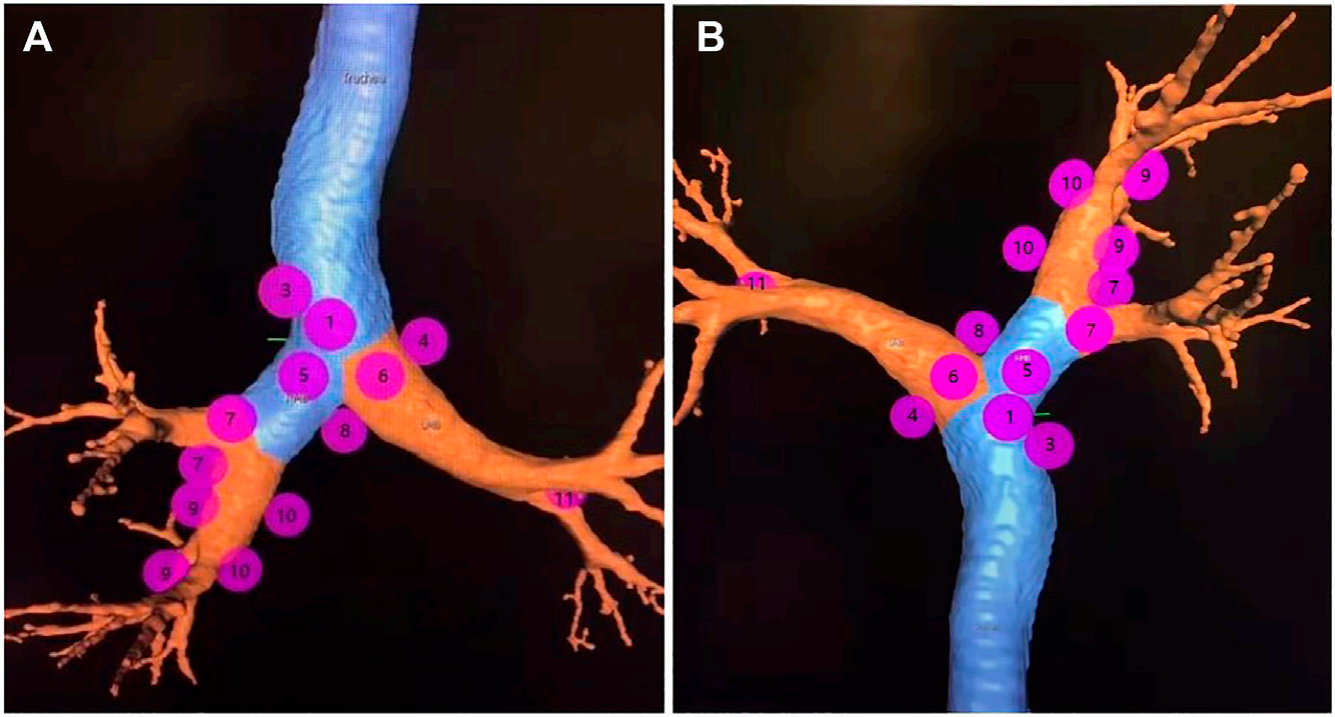
Note: The overall view of eleven lymph node stations of Wang’ map in a 3D reconstructed imaging.

**FIGURE 2:**
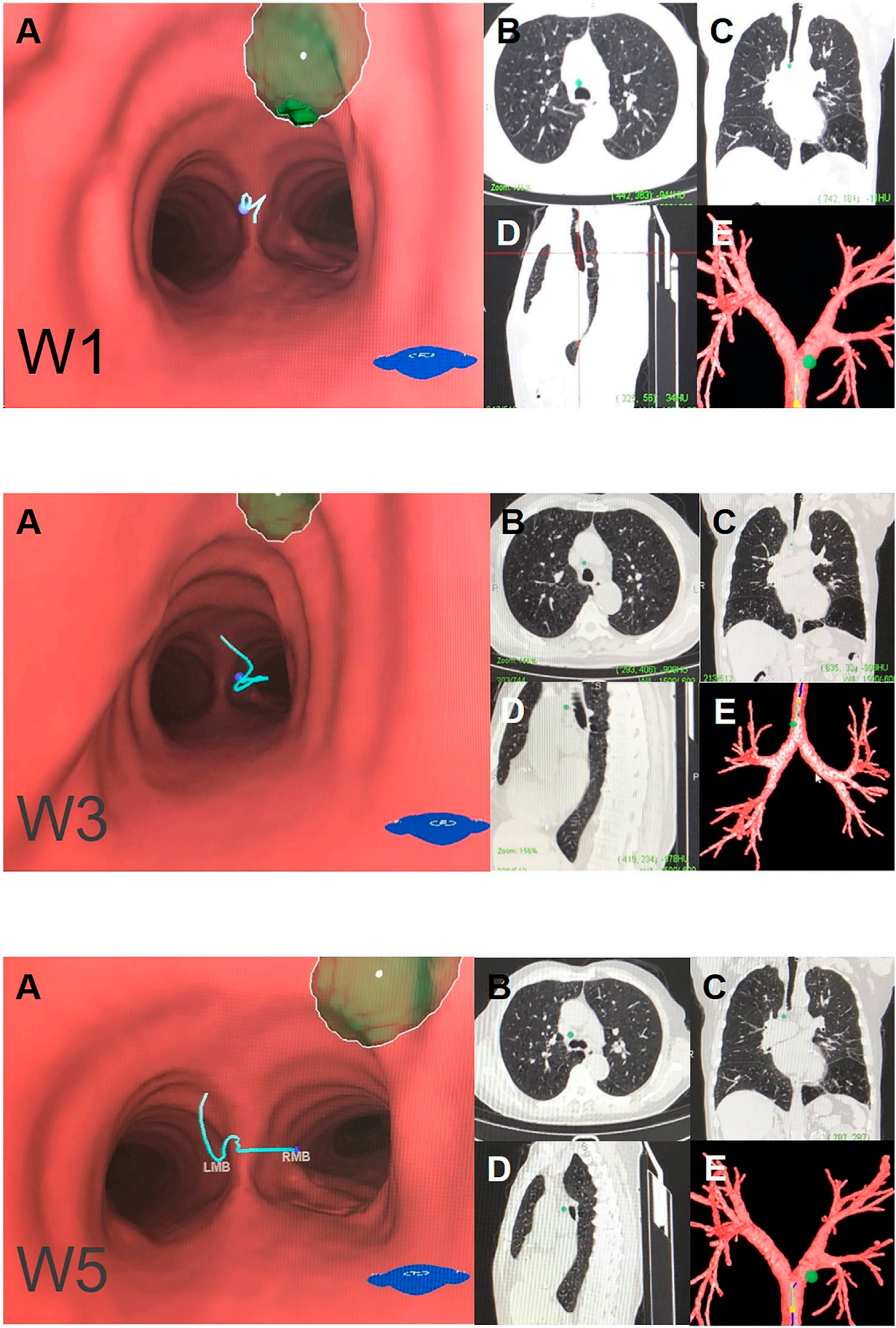
Right mediastinal lymph node (N2) Note: (W1) anterior carina lymph nodes. (W3) right paratracheal lymph nodes. (W5) right main bronchus lymph nodes. **(A)** Intraluminal view and puncture sight (green). **(B)** Axial view. **(C)** Coronal view. **(D)** Sagittal view. **(E)** Reconstruction image.

**FIGURE 3:**
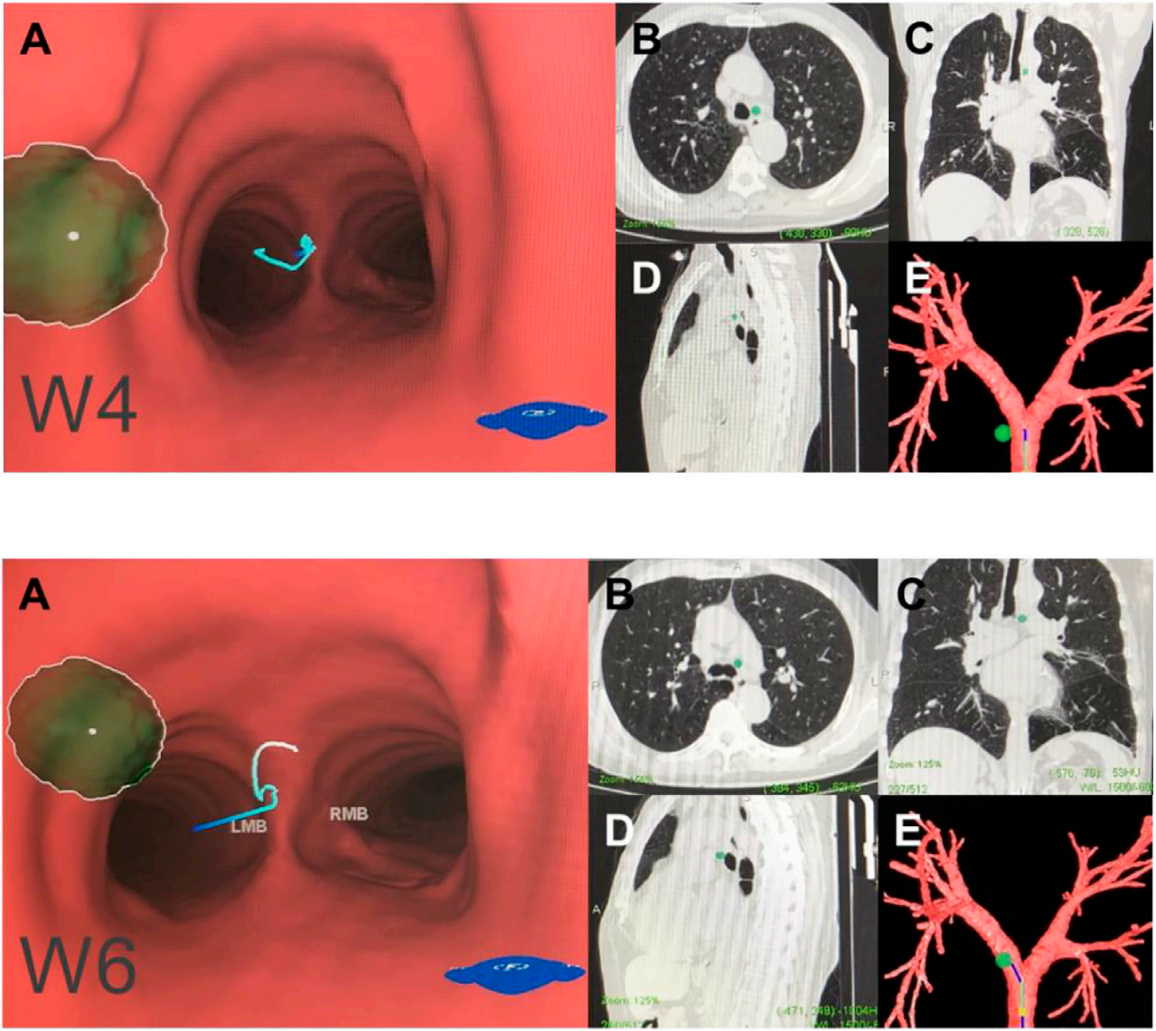
Left mediastinal lymph node (N2). Note: (W4) left paratracheal lymph nodes or aortic pulmonary (A-P) window lymph nodes. (W6) left main bronchus lymph nodes. **(A)** Intraluminal view and puncture sight (green). **(B)** Axial view. **(C)** Coronal view. **(D)** Sagittal view. **(E)** Reconstruction image.

**FIGURE 4:**
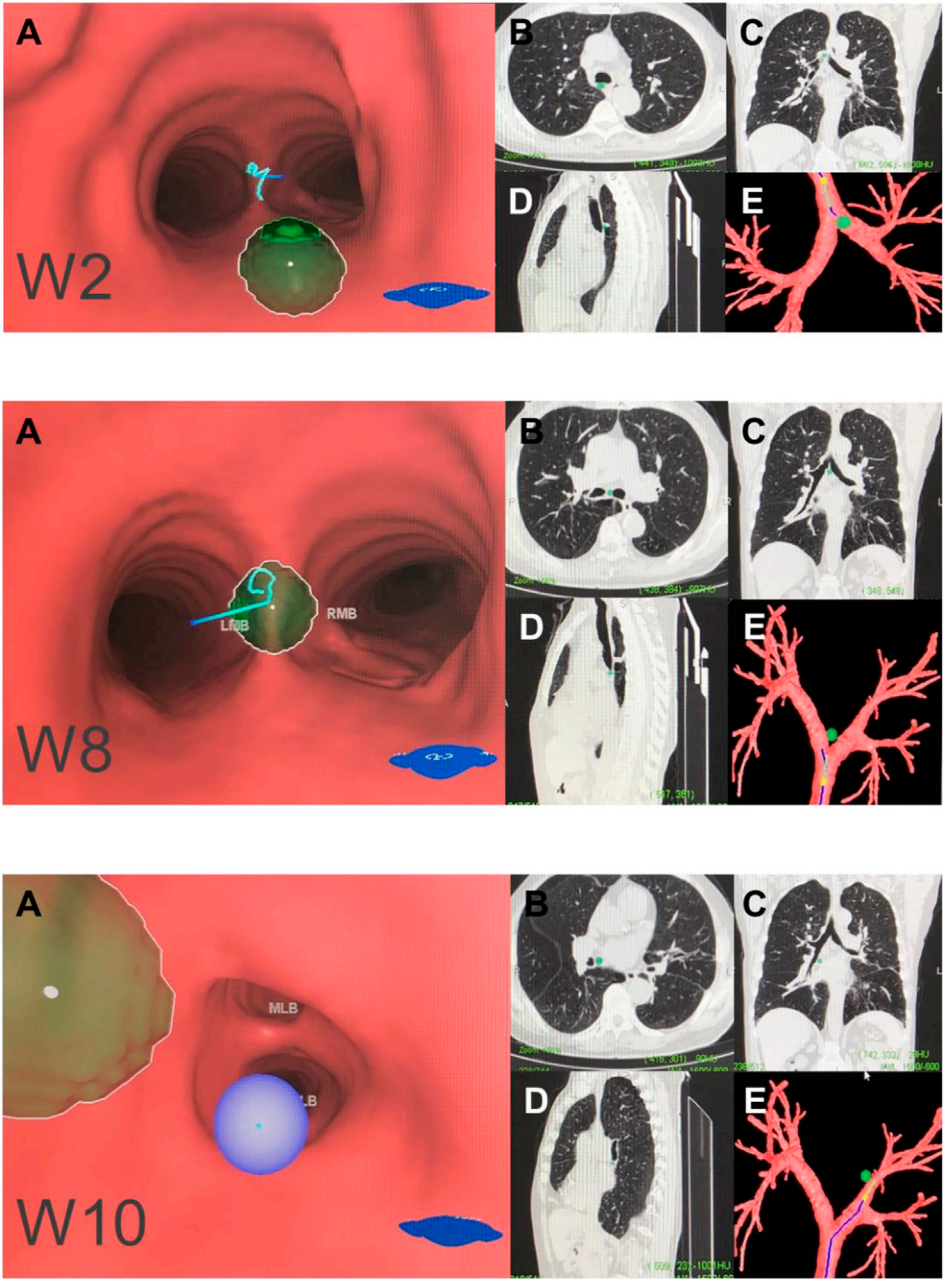
Central mediastinal lymph node (N2). Note: (W2) posterior carina lymph nodes. (W8) subcarinal lymph nodes. (W10) subsubcarinal lymph nodes. **(A)** Intraluminal view and puncture sight (green). **(B)** Axial view. **(C)** Coronal view. **(D)** Sagittal view. **(E)** Reconstruction image.

**FIGURE 5:**
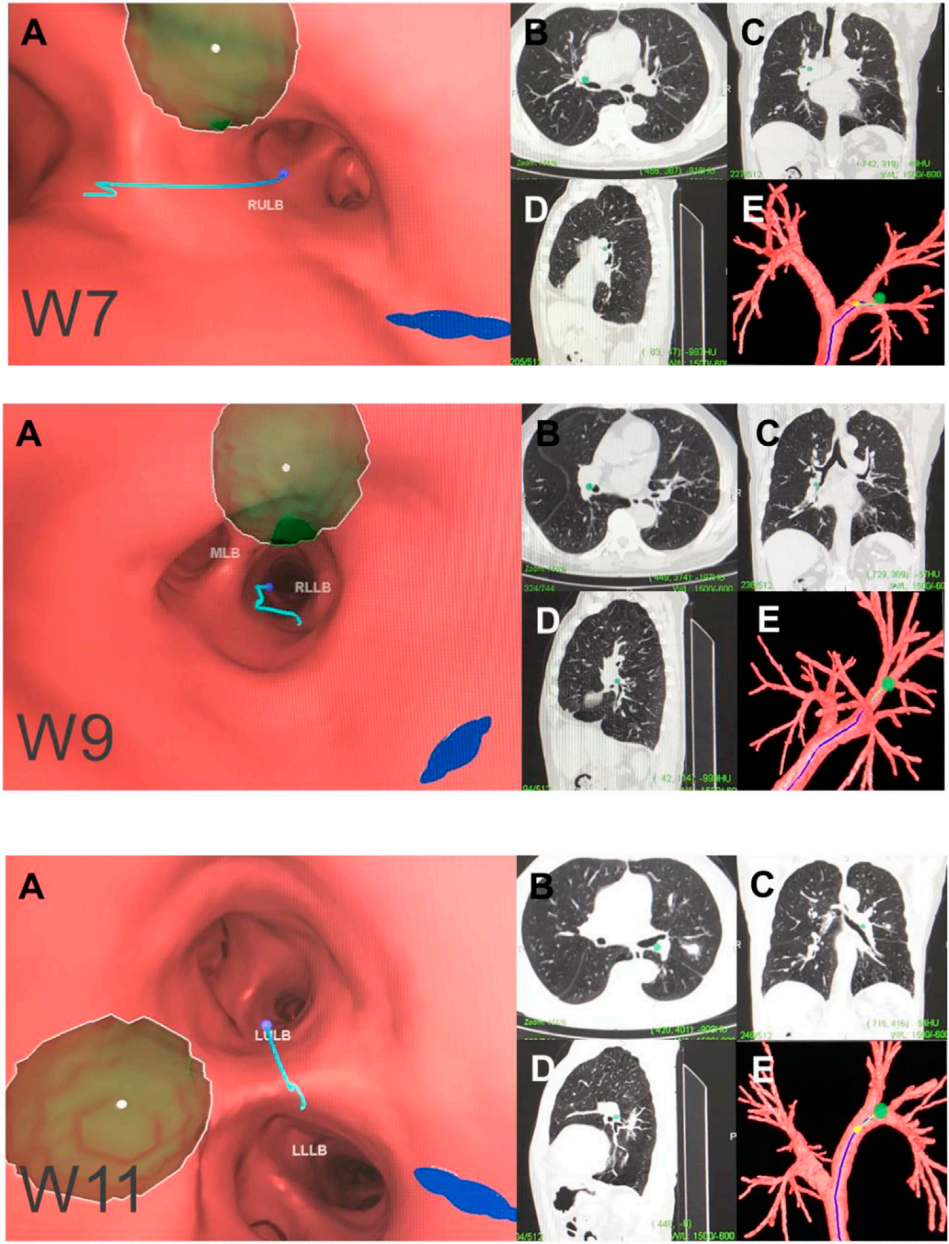
Hilar lymph node (N1). Note: (W7) right upper hilar lymph nodes. (W9) right lower hilar lymph nodes. (W11) left hilar lymph nodes. **(A)** Intraluminal view and puncture sight (green). **(B)** Axial view. **(C)** Coronal view. **(D)** Sagittal view. **(E)** Reconstruction image.

We generated an overall view of 11 lymph node stations in Wang’ map under 3D reconstruction (Figure 1). Next, we collected the horizontal, coronal and sagittal views of chest CT, labeled extrabronchial 3D anatomical locations of the 11 nodal stations in terms of Wang’s lymph node map and generated a virtual view from an extrabronchial perspective. We classified the 11 stations into four groups: right mediastinal stations (Figure 2), left mediastinal stations (Figure 3), central mediastinal stations (Figure 4) and hilar stations (Figure 5). The recommended TBNA puncture sites of particular lymph nodes were also described in detail (Wang, 1994; Wang, 1995; Xia and Wang, 2013) (see Supplementary Table S1).

W1 station, anterior carina (correlated with IASLC 4R).
Location: In front and between the proximal portions of the right and left main bronchi.
Posterior border: The carina of the trachea.
Puncture point: The first trachea cartilage ring gap at 12–1 o’clock.
W2 station, posterior carina (correlated with IASLC-7).
Location: Behind and between the proximal portions of the right and left main bronchi or directly behind the right main bronchus.
Upper border: tip of the carina.
Lower border: The superior portion of the opening of the right upper lobe bronchus.
Puncture point: The opposite side of W1, 5–6 o’clock at the posterior wall of the carina.
W3 station, right paratracheal (correlated with IASLC-4R).
Location: Behind the superior vena cava and in front of the anterolateral aspect of the lower trachea near the azygous arch.
Upper border: The inferior margin of the brachiocephalic vein or superior margin of the aortic arch.
Lower border: The superior margin of the azygous arch.
Puncture point: Second to fourth trachea cartilage ring gaps at 1–2 o'clock.
W4 station, left paratracheal (A-P window, correlated with IASLC-4L).
Location: Lateral to trachea near the tracheobronchial angulation, below the aortic arch and above the left main pulmonary artery.
The W4 lymph nodes are divided into interior, middle and exterior windows.
Puncture point: First or second cartilage ring gap from the distal trachea at 9 o'clock or one trachea cartilage ring distal or proximal from the tracheobronchial angle.
W5 station, right main bronchus (distal W5 correlated with IASLC-10R, proximal W5 correlated with IASLC-4R).
Location: In front of the proximal proportion of the right main bronchus.
Puncture point: The first or second trachea cartilage ring gap of the right main bronchus at 12 o'clock.
W6 station, left main bronchus (correlated with IASLC-10L).
Location: In front of the proximal proportion of the left main bronchus. The W6 lymph node is a mediastinal lymph node.
Puncture point: The first or second trachea cartilage ring gap of the left main bronchus at 12 o'clock.
W7 station, right upper hilar (correlated with IASLC-11Rs).
Location: In front and between the right upper lobe bronchus and bronchus intermedius.
Puncture point: Anterolateral direction of the right upper lobe crest.
W8 station, subcarina (correlated with IASLC-7).
Location: Between the right and left main bronchi, at or near the level of the right upper lobe bronchus.
Upper border: The superior margin of the opening of the right upper lobe bronchus.
Lower border: The opening of the right intermediate bronchus.
Puncture point: Right main bronchus at the level of the opening of the right upper lobe bronchus, at 9 o'clock.
W9 station, right lower hilar (correlated with IASLC-11Ri).
Location: Lateral or in front of the bronchus intermedius, at or near the level of the right middle lobe bronchus. The W9 lymph nodes consist of lymph nodes at the right lateral side of the right intermediate bronchus and near the ridge of the right middle and lower lobe bronchi.
Puncture point: Lateral side of the right intermediate bronchus.
W10 station, subsubcarina (correlated with the lower part of IASLC-7).
Location: Between the bronchus intermedius and the left main bronchus, at or near the level of the right middle lobe bronchus.
Upper border: The opening of the right intermediate bronchus.
Lower border: The distal end of the right intermediate bronchus, extending to the inferior side of the opening of the right middle lobe.
Puncture point: Right intermediate bronchus at 9 o'clock.
W11 station, left hilar (correlated with IASLC-11L).
Location: Between the left upper lobe and the left lower lobe bronchus.
Puncture point: The opening of the dorsal segment of the left lower lobe at 9 o'clock.

## Discussion

In the present study, we introduced a virtual map of lymph node landmarks under the assistance of a VBN system. Our map displays the intrabronchial and extrabronchial spatial relationships of 11 lymph node stations and their recommended puncture sites corresponding to Wang’s map.

The VBN system in combination with CT of the 11 mediastinal and hilar lymph nodes and their surrounding structures from extrabronchial and endobronchial, sagittal, coronal as well as horizontal views provided a full understanding of the thoracic lymph nodes and their intrabronchial and extrabronchial relationships with adjacent tissues and blood vessels. A detailed view of the lymph nodes and their adjacent tissues can reinforce the understanding of the anatomy and assist in training of operators for TBNA.

There are two major differences between Wang’s map and the IASLC map (Xia et al., 2015). First, stations five and six in Wang’s map are located in front of the proximal proportion of the right and left main bronchi, respectively. Both W5 and W6 stations are defined as mediastinal lymph nodes. Second, W5 covers two IASLC lymph nodal stations: the distal end of 4R as well as 10R station. Third, compared with the previous edition of the IASLC lymph node map, the lower border is extended to the azygos vein on the right, which fits the principle of Wang’s map that 4R is correlated with station W1 and W3 as well as proximal W5 station. Similarly, the lower border of station seven in the IASLC map is extended to the upper border of the lower lobe bronchus on the left and the lower border of the bronchus intermedius on the right. This changed definition is also correlated with the definitions of W8 and W10.

VBN is generally conducted for guidance in treatment of PPLs. PPLs cannot be accessed easily by cTBNA because they are distinct from the trachea and central bronchus. The development of image-guided bronchoscopy techniques significantly enhances the diagnostic accuracy of PPLs. The VBN system was designed to reach and guide aspiration of PPLs with a diagnostic value of 72.0%–73.8% (Wang Memoli et al., 2012; Asano et al., 2014), and the diagnostic value reached 67.4% for lesions <2 cm in diameter (Asano et al., 2014). In combination with CT-guided ultrathin bronchoscopy, the diagnostic value of VBN increases to 65.4%–81.6% (Asano et al., 2014). On the other hand, the VBN system has also been applied in diagnosis of thoracic lymph node adenopathy. The sensitivity and diagnostic accuracy are both significantly increased for mediastinal lymph nodes by VBN (McAdams et al., 1998; Fiorelli et al., 2017), suggesting that the VBN system is also valuable in cases of mediastinal lymph node enlargement.

Aside from the advantages of the VBN system, the bronchoscopist still has to perform cTBNA blindly in clinical practice, as the exact puncture site cannot be confirmed in real-time. In general, the main purpose of generating our multi-dimensional virtual Wang’s lymph node map is for training junior interventional pulmonology fellows or bronchoscopists. Compared with EBUS-TBNA, our approach does not require the use of a regular scope for the airway survey. In addition, although EBUS-TBNA allows real-time visualisation of a punctured lymph node, the operator cannot precisely identify the puncture site. In contrast, our system probes the 3D anatomy of each lymph node station with the puncture site. We expect that our multi-dimensional virtual Wang’s lymph node map would allow precise targeting of lymph nodes 1–2 cm in diameter. Hence, the two methods are not competitive but complementary for bronchoscopists within the learning curve. The diagnostic yield is satisfactory upon combining virtual endobronchial ultrasound with EBUS-TBNA bronchoscopy when diagnosing mediastinal-hilar lymph node enlargement (Sato et al., 2013), but its necessity is doubtful. In addition, rapid on-site evaluation (ROSE) may be a good alternative to EBUS for combination with the VBN system. In addition, we recommend a probe designed to determine whether the needle is aligned in the puncture site. When it is aligned properly, the inducer could turn from green to red to alert the practitioner of puncture. Such a device may further improve the diagnostic value of VBN-guided TBNA.

There was a significant limitation of this study in that the diagnostic yield of this particular VBN-assisted TBNA has not yet been verified. A clinical trial is required to examine the validity of this VBN plus TBNA system. In addition, the quality of the CT image can also affect the accuracy of the derived 3D images.

Overall, we have proposed a virtual map for intra- and extrabronchial landmarks of hilar and mediastinal lymph nodes based on the Lungpoint VBN system. This map can help in training bronchoscopists as well as enhancing the diagnostic value of the VBN system. Further clinical trials are required to determine the efficacy of this strategy.

## Data Availability

The original contributions presented in the study are included in the article/Supplementary Material, further inquiries can be directed to the corresponding authors.
