# Supplementary material for: Multi-Dimensional Display of Wang’s Lymph Node Map Using Virtual Bronchoscopic Navigation System
Source: Front Mol Biosci. 2021 Jun 7;8:679442. doi: 10.3389/fmolb.2021.679442 (PMC8215157; doi:10.3389/fmolb.2021.679442)
Supplement: Supplementary file 1 [file Table1.DOC]

**Comparison of the Wang’s and IASLC lymph node maps with respect to the anatomical definitions**.

| **Wang’s Map** | **Puncture sites for TBNA** | **IASLC** |
| --- | --- | --- |
| Right mediastinal lymph node (N2) | | |
| **Station 1 lymph nodes (W-1):** named anterior carina lymph nodes, are in front of the intersection of the left and right main bronchi. | The lymph nodes can be punctured and sampled at about the 12 o’clock position between the first and second tracheal cartilage rings from the lower trachea. | **#4 Lower Paratracheal Nodes**  **4R**: includes right paratracheal nodes, and pretracheal nodes extending to the left lateral border of trachea  Upper border: intersection of caudal margin of innominate vein with the trachea  Lower border: lower border of azygos vein |
| **Station 3 lymph nodes (W-3)**: named as right paratracheal lymph nodes, are above the azygous arch, and in front of the anterolateral to lower trachea. The lymph nodes range from the lower border of the brachiocephalic vein or the upper border of the aortic arch to the upper margin of the azygous arch. | The lymph nodes can be punctured and sampled at about the 1-2 o’clock position between the second and fourth tracheal cartilage rings from the lower trachea |
| **Station 5 lymph nodes (W-5)**: named the right main bronchus lymph nodes, are in front of the proximal right main bronchus. | The lymph nodes can be punctured and sampled at about the 12 o’clock position in the first or second intercartilaginous interspace from the proximal right main bronchus. |
| Left mediastinal lymph node (N2) | | |
| **Station 4 lymph nodes (W-4)**: named left paratracheal lymph nodes or aortic pulmonary (A-P) window lymph nodes, are left lateral to the trachea near the tracheobronchial angulation, or at the left side of the proximal left main bronchus or tracheobronchial angulation. Lymph nodes between the aortic arch and left pulmonary artery are also called A-P window lymph nodes, and include all lymph nodes medial or lateral to the ligmentum arteriosum. | These lymph nodes can be punctured and sampled at about the 9 o’clock position in the first or second intercartilaginous interspace from the left side of the lower trachea. | **#4 Lower Paratracheal Nodes**  **4L:** includes nodes to the left of the left lateral border of the trachea, medial to the ligamentum arteriosum  Upper border: upper margin of the aortic arch  Lower border: upper rim of the left main pulmonary artery |
| **Station 6 lymph nodes (W-6)**: named left main bronchus lymph nodes, are in front of the proximal left main bronchus. | The puncture sites for left main bronchus lymph nodes are at about the 12 o’clock position between the first and second tracheal cartilage rings from the proximal left main bronchus. |
| Central mediastinal lymph node (N2) | | |
| **Station 2 lymph nodes (W-2)**: named posterior carina lymph nodes, are behind the intersection of the left and right main bronchi, or directly behind the right main bronchus. The lymph nodes range from the intersection of the left and right main bronchi to the upper border of the right upper lobe bronchus orifice on CT imaging. | The lymph nodes can be punctured and sampled in the posterior portion of the carina at about the 5-6 o’clock position, and are sometimes closer to the right main bronchus. | **#7 Subcarinal Nodes**  Upper border: the carina of the trachea  Lower border: the upper border of the lower lobe bronchus on the left; the lower border of the bronchus intermedius on the right |
| **Station 8 lymph nodes (W-8)**: named subcarinal lymph nodes, are between the right and left main bronchi, ranging from the upper border of the right upper lobe of the bronchus orifice to the bronchus intermedius orifice. When the right upper lobe bronchus orifice can be seen, the lymph nodes are categorized as subcarinal lymph nodes according to the corresponding CT scan image. | The puncture sites for subcarinal lymph nodes are at about the 9 o’clock position in the medial wall of the right main bronchus, proximal to the right upper lobe orifice. If the lesion is punctured inward and backward, the needle tip can reach the level of the orifice of the right upper lobe of the bronchus. |
| **Station 10 lymph nodes (W-10):** named subsubcarinal lymph nodes, are between the bronchus intermedius and left main bronchus, ranging from the upper to the lower borders of the bronchus intermedius. | The puncture sites for subsubcarinal lymph nodes are at about the 9 o’clock position in the medial wall of the bronchus intermedius. Subsubcarinal lymph nodes are widely distributed, and can be punctured and sampled at the upper, middle and lower levels of the bronchus intermedius. |
| Hilar lymph node (N1) | | |
| **Station 7 lymph nodes (W-7):** named right upper hilar lymph nodes in Wang’s map, are in front of and between the right upper lobe and the bronchus intermedius. | The puncture sites for the right upper hilar lymph nodes are at the anterior portion of the right upper bronchus ridge. | **#11 Interlobar Nodes**  **11Rs:** between the upper lobe bronchus and bronchus intermedius on the right |
| **Station 9 lymph nodes (W-9):** named right lower hilar lymph nodes in Wang’s map, are located in the area beside the bronchus intermedius, and include all lymph nodes beside the bronchus intermedius and near the ridge of the right middle lobe and right lower lobe. | The puncture sites for the right lower hilar lymph nodes are at the lateral or anterior wall of the bronchus intermedius at about the 3 or 12 o’clock position, near or at the level of the right middle lobe orifice. | **11Ri:** between the middle and lower lobe bronchi on the right |
| **Station 11 lymph nodes (W-11):** named left hilar lymph nodes, are between the left upper lobe and left lower lobe of the bronchus. | The puncture sites for left hilar lymph nodes are at the lateral wall of the left lower lobe of the bronchus at about the 9 o’clock position, proximal to the left dorsal bronchus orifice. | **11L**: between the origin of the lobar bronchi on the left. |
